# Empathy and Self-Efficacy in Elderly Nursing Practice among Korean Nurses

**DOI:** 10.3390/ijerph18063072

**Published:** 2021-03-17

**Authors:** Seyoon Kim, Hyun Jin Roh, Sohyune Sok

**Affiliations:** 1 Department of Nursing, Graduate School, Kyung Hee University, Seoul 02447, Korea; ssamyun85@naver.com (S.K.); angelnhj@nate.com (H.J.R.); 2 College of Nursing Science, Kyung Hee University, 26, Kyungheedae-ro, Dongdaemun-gu, Seoul 02447, Korea

**Keywords:** aged, empathy, self-efficacy, nursing

## Abstract

The Korean government is activating an integrated nurse care service, in which all inpatient care services are provided by a proper nursing team without a caregiver or a guardian staying at the hospital. This study was to examine the influence of empathy and self-efficacy on the elderly nursing practice of nurses at integrated nurse care service wards in South Korea. A cross-sectional descriptive design was employed. Participants included 238 nurses who were working at integrated nurse care service wards in hospitals in Seoul, South Korea. Measures were a general characteristics form, the Empathy Construct Rating Scale, the Korean Self-Efficacy Scale, and the Korean Elderly Nursing Practice Scale. Data were collected from February to May 2017. The findings show a slightly higher level of empathy; an almost intermediate level of self-efficacy; and a high level of elderly nursing practice in Korean nurses at integrated nurse care service wards. The only factor found to impact significantly on elderly nursing practice was empathy. The findings suggest that nurses need to enhance empathy toward the elderly to improve elderly nursing practice. It is necessary to provide strategies or interventions in the nursing management for improvement of the empathy of nurses at integrated nurse care service wards in South Korea.

## 1. Introduction

In South Korea, the proportion of the elderly aged 65 years or older is expected to exceed 40% by 2058, from 15.7% in 2020 [1]. As the number of elderly inpatients has increased due to population aging and increased chronic diseases, the Korean government launched a caregiverless hospital project in 2013 to provide care services by nurses, reduce the family burden on taking care of a patient, and improve the inpatient services [2]. Since then, the program has developed into an integrated nurse care service, in which all inpatient care services are provided by a proper nursing team without a caregiver or a guardian staying at the hospital [2]. The program was be expanded to include all hospitals from 2018 [2]. The proportion of the elderly aged 65 years or older who utilized the integrated nurse care service has gradually increased, from 37.2% in 2013 to 49.6% in 2019 [3].

Due to complex health issues and a lack of understanding, the nurses feel more stressed with regard to taking care of elderly patients [4,5,6]. It is important that the nurses communicate with the elderly patients according to their ability to understand [4,7,8]. For this reason, empathy is an essential element in the relationship between nurses and elderly patients [4,9]. Empathy is a form of communication that reacts according to the emotions of the patients, thus positively affecting the recovery of the elderly [10,11]. It is the key factor in high-quality elderly nursing practice [8,12]. Elderly care is a mutual process between the nurses and elderly patients that provides a solution for the discomfort, personal hygiene, and health problems caused by aging [9,11]. It is very important to identify uncommon nursing problems that the elderly have based on knowledge and attitude, and to provide nursing care in order to solve these issues [5,13]. According to previous studies, the elderly showed deficits in empathic thinking as their cognitive abilities decreased [4,7,8], and empathy was significantly correlated with elderly nursing practice, as compared to the clinical nurses’ attitude toward the elderly or their experiences [5,9,13].

As the indirect nursing practice area, which manages and supervises a certain part of the family, and caregivers, who provide nursing and care, have been included in the direct nursing practice area due to the implementation of the integrated nurse care service [14], the nurses feel more stressed about performing nursing duties in a work environment different from the previous one, thereby leading to a deterioration in the quality of nursing [9,15]. The integrated nurse care service meets the demands of the times because it is considered a change in the nursing paradigm, and a system that solves the problems of the current medical environment. However, it requires nurses to adapt to the increased workload and the new environment, and it is necessary to make efforts to stabilize the system [5]. For this reason, self-efficacy is important as it allows an individual to judge his or her ability to organize and perform the actions needed to accomplish a specific task [16]. The higher the self-efficacy, the more positive the influence on the nursing performance, thus leading to higher quality nursing [5,17]. The integrated nurse care service ward (INCSW) provides direct nursing, along with prompt, accurate, and professional knowledge [3,8]. Such self-efficacy is expected to have a positive effect on elderly care, thereby helping nurses to rapidly adapt to the new nursing environment [5,9]. Therefore, this study was conducted to determine the influence of empathy and self-efficacy on elderly nursing practice in nurses who are working at an integrated nurse care service ward, and offer basic data for improving the quality of elderly care. The aims of this study were: (1) to examine the levels of empathy, self-efficacy, and elderly nursing practice; (2) to examine the differences in elderly nursing practice according to general characteristics; and (3) to examine the influential factors relating to elderly nursing practice.

## 2. Material and Methods

### 2.1. Study Design and Participants

This study used a cross-sectional descriptive design. Participants included 210 nurses who were working at integrated nurse care service wards in hospitals in Seoul, South Korea. Study participants participated through random sampling in this study. The eligibility criteria were: over 20 years old; working for 6 months or over for elderly patients at an integrated nurse care service ward; recognition of research objectives; willingness to participate; and able to communicate in Korean. There were no special exclusion criteria. Of the 250 questionnaires distributed, 243 (97.2%) were returned. Data from several questionnaires were incomplete, so data from 238 questionnaires (97.9%) were included in the final dataset. Sample size suitability (*n* = 146) using the F test and G power 3 analysis software was estimated based on an alpha level of 0.05, a medium effect size of 0.15, and a power of 0.95 [18]. Hence, the sample size was adequate.

### 2.2. Measures

Based on a literature review and previous research, a set of study participants’ general characteristic variables, including gender, age, educational level, religion, marital status, total working experience, working experience of an integrated nurse care service ward (INCSW), department ward, job title, satisfaction with current workplace, level of salary, experience of living with the elderly, and experience in psychiatric ward work, were obtained via self-report. This consisted of a total of 13 items. 

The Empathy Construct Rating Scale (ECRS), developed by Monica [19], was revised by Park, Suk, and Jung [20] and used to investigate empathy. This tool consisted of 60 items. A 5-point Likert scale was used with the score ranging from 60 to 300 points, and the higher the score, the higher competence in empathy. ECRS showed an acceptable to content validity in the previous study [20]. Reliability in this study was Cronbach’s α = 0.83.

The Korean Self-Efficacy Scale, developed by Kim and Cha [21], was used to find out the subject’s self-efficacy. This tool consisted of 24 items in total. A 5-point Likert scale was used with the score ranging from 24 to 120 points, and the higher the score, the higher the self-efficacy. In this study, reliability was Cronbach’s α = 0.85.

The Korean-Elderly Nursing Practice Scale, developed by Choi [22] was used to investigate elderly nursing practice. This tool consisted of 16 items in total. A 4-point Likert scale was used with the score ranging from 16 to 64 points, and the higher the score, the higher the elderly nursing practice. In this study, reliability was Cronbach’s α = 0.85.

### 2.3. Procedures

The researcher contacted 5 hospitals with integrated nurse care services in Seoul through e-mails. Hospitals with integrated nurse care service wards were visited to obtain the permission for this study. Participants were randomly selected using the coin toss. Researchers contacted the prospective study participants in personal meetings and explained the purpose of this study, as well as participation details and the questionnaire used. The researchers received written informed consent forms from study participants who agreed to participate in this study. The questionnaires were given only to study participants who agreed to participate in the study. After that, the completed questionnaires were collected. The survey consisted of a self-reporting questionnaire, managed by researchers. Each questionnaire took about 25 min to complete. Data were collected from February to May 2017.

### 2.4. Statistical Analysis

Data were analyzed by the SPSS PC+ version 21.0 statistical software program (IBM Corp., Armonk, NY, USA). The subjects’ general characteristics and the study variables’ level were examined by descriptive statistics. Correlations among the study variables were probed by the Pearson correlation coefficient. Differences in elderly nursing practice according to general characteristics were examined by *t*-test and F test. Multiple regression analysis was used to examine the factors influencing elderly nursing practice. A statistically significant consideration was a *p*-value of less than 0.05.

### 2.5. Ethical Considerations 

After obtaining the approval of the Institutional Review Board (KHSIRB-17-003), we revealed that the data would not be used for purposes other than research by obtaining voluntary cooperation on data collection from the subjects. In addition, the subjects were informed that they could withdraw from the study at any time. They were also informed of the anonymity and confidentiality of the data obtained from them. Researchers obtained the completed written consent forms from the selected study participants. The collected questionnaires of the subjects will be discarded by using a document shredder after the research paper is finally published.

## 3. Results

### 3.1. General Characteristics of Study Participants

As general characteristics of the participants, women (95.8%) were the primary gender and the average age of the participants was 29.34 years old. The largest age group was below 30 years of age (52.6%). Slightly below half (48.2%) of the participants had a bachelor’s as their level of education, followed by a college education (45.5%). Most were atheists (55.2%) and single (77.3%). The total working experience of participants was mostly below 5 years (57.8%). The average working experience on an INCSW was 1.97 years. In terms of department ward, surgical ward was the most common (43.3%), followed by internal medicine (32.8%). As for satisfaction with current workplace, most were moderately satisfied (54.2%), and only 37.8% had experience of living with the elderly. Most (96.2%) had no experience in psychiatric ward work (Table 1).

### 3.2. Levels of Empathy, Self-Efficacy, and Elderly Nursing Practice

The mean score of the participants for empathy was 192.12 (SD = 14.86), indicating a slightly higher level of empathy as compared with the median value (180 points); the mean score for self-efficacy was 76.23 (SD = 7.42), showing an almost intermediate level of self-efficacy as compared with the median value (72 points); and the mean score for elderly nursing practice was 52.78 (SD = 7.62), exhibiting a high level of elderly nursing practice as compared with the median value (40 points) (Table 2).

### 3.3. Correlations among the Study Variables 

Analysis of the correlation between elderly nursing practice and empathy showed a positive correlation (r = 0.27, *p* < 0.001). In terms of the correlations between empathy and related factors, the analyses of age (r = 0.17, *p* < 0.001) and total working experience (r = 0.16, *p* < 0.05) showed a positive correlation. Analysis of the correlation between self-efficacy and age showed a positive correlation (r = 0.13, *p* < 0.05) (Table 3).

### 3.4. Differences in Elderly Nursing Practice According to the General Characteristics of Study Participants

There was a significant difference between levels of experience in psychiatric ward work (*t* = 2.48, *p* = 0.023). There were no significant differences linked to other general characteristics (Table 4).

### 3.5. Influential Factors of Elderly Nursing Practice

In order to determine whether this study was suitable for regression analysis, the assumptions of the regression model were tested. First, homoscedasticity was confirmed based on the result of examining the residual plot. The result of testing the autocorrelation of the error with Durbin–Watson to verify the independence of the residuals shows that they all met the regression assumption without autocorrelation, as the test statistic was 1.78, which was between 1.69 and 1.89. In addition, the tolerance of multicollinearity was from 0.218 to 0.852, which was more than 0.10, and the variance inflation factor (VIF) was from 1.143 to 1.652. Since the VIF value was not greater than 10, all variables were found to have no problems of multicollinearity in this study. 

In the multiple-regression analyses, the main variables, such as empathy, self-efficacy, and elderly nursing practice, were inputted with the general characteristics, including participant marital status, total working experience, job title, and experience of living with the elderly. The regression model was statistically significant in this study (F = 48.89, *p* < 0.001). The explanatory power of the regression model was 32%. The statistically significant variable in the regression model was empathy (β = 0.30) (Table 5).

## 4. Discussion

The average age of the subjects was 29.34 years old, and most of them were in their 20s (52.6%). They had been working at an integrated nurse care service ward for 1.97 years on average. In the study conducted by Yeun [23] on nurses who were working at an integrated nurse care service ward, nurses who were between 25 and 29 years old formed the majority, at 61.4%. The reason for the lower average age of these nurses is that the ratio of patients to nurses had decreased due to the nature of the ward as compared to before. Therefore, new nursing staff seem to be employed according to the increased demand for more nursing personnel.

In this study, the level of empathy of the nurses was lower than that in the studies conducted by Song [8] and Seo et al. [13], which used the same tool, and the level of empathy was particularly high in a study conducted on psychiatric ward nurses [24]. Elderly patients may show weakness in empathic thinking, as compared to patients in other age groups, due to the lowered cognitive function [7,11,13]. Since empathy is interactive, it is inferred that the low empathy of the elderly patients may have affected the nurses who were taking care of them [5,8,11]. In addition, it is thought that the low empathy of nurses toward the elderly patients was due to the fact that the average age of the nurses working at the integrated nurse care service wards was lower and the age difference was greater.

The level of self-efficacy was lower than that of a study conducted on the general ward nurses using the same tool [17]. Based on the results of this study, which show a positive correlation between self-efficacy and age, most nurses at the integrated nurse care service wards are young and they have relatively less clinical experience. This seems to be the reason that their level of self-efficacy was low. Due to the nature of the ward, the roles and duties of the nurses were increased, along with an increase in direct nursing hours, and their self-esteem was lowered because they had to perform the duties that had been done by caregivers [25,26]. Moreover, the lack of clarity in the division of work among the nurses, and the system that accepts inpatients regardless of the severity of disease and the disease group, or without distinguishing between internal medicine and surgery patients, also resulted in confusion and an overload of work, which seemed to be the cause of low self-efficacy. 

Though there is no study on the degree of elderly nursing practice among the nurses who are providing the nursing care integration service, the degree of elderly nursing practice in this study was lower than that of the studies conducted on the nurses in elderly nursing care centers using the same tool [27,28]. The reason seems to be that the nurses in the elderly care centers, where nursing is performed only for the elderly, are more dedicated to practical tasks for the elderly patients [25,26]. Although most of the patients in the wards operated under the system of integrated nurse care service are elderly, acute care and disease-centered treatment is mainly performed, and there is no age limit for the target patients, which seems to have resulted in a relatively low score in the degree of elderly nursing practice. Moreover, it would have been difficult to offer elderly care that was suitable for the target patients due to the increase in direct nursing load.

In terms of the correlations among the study variables, empathy had a positive correlation with elderly nursing practice, while empathy and self-efficacy had positive correlations with age. These results show that the higher the empathy of the nurses toward the elderly patients, the higher their competence in elderly nursing practice; and the older they are, the higher empathy and self-efficacy they have. Furthermore, the more clinical experience that the nurses have, the more empathy they have, thereby showing that nurses with more clinical experience exhibit more empathy. These results are identical to those of previous studies [5,6,8,9,13,27].

In regard to the differences in the degree of elderly nursing practice according to the general characteristics of the study participants, the degree of elderly nursing practice was significantly higher when the nurses had experience in psychiatric wards, as compared to those without psychiatric ward experience. This result supports those of previous studies [7,13,24], thus showing that the nurses with psychiatric ward experience have higher empathy toward the patients. 

Empathy toward the elderly patients is shown as a predictor that significantly affects elderly nursing practice. In order to improve the competence at elderly nursing practice, empathy is an antecedent, and it was found that a high degree of empathy significantly affected elderly nursing practice, which is consistent with the results of previous studies [5,9,13].

Based on the study findings, we recommend that the integrated nurse care service wards require nurses with enough clinical experience to understand the characteristics of elderly care and provide appropriate care. Nurses who care for the elderly patients need to respond sensitively to their physical, emotional, and social needs, as well as closely monitor the behaviors and expressions of the elderly patients, and understand and empathize from different perspectives. Future research must expand the number of subjects and conduct repetitive studies. Furthermore, various experimental studies are required in order to develop different programs to enhance empathy toward the elderly among the nurses in the integrated nurse care service wards, and to verify their effectiveness. 

This study has some limitations. This study is limited in that the nurses, who were working at general hospitals in Seoul, were selected as study subjects via convenience sampling. For this reason, caution should be taken in generalizing the results. In regard to hospitals that are operating the integrated nurse care service, the workload, hospital characteristics, and the situation by ward in each hospital could not be fully considered, as patients were admitted without any restrictions on the severity of their condition and disease group. 

## 5. Conclusions

In conclusion, this study found that the higher the level of empathy of the nurses at the integrated nurse care service wards, the higher the degree of their elderly nursing practice. Such empathy was confirmed to have a statistically significant effect on the degree of elderly nursing practice. Since more integrated nurse care service wards will be established, the nurses must respond sensitively to the various demands of the elderly patients, monitor them carefully, try to understand the elderly patients, and empathize with them. Therefore, in order to provide high quality elderly nursing practice, it is necessary to have psychological support programs for the nurses, mentoring by more experienced nurses, and psychological support to build the level of empathy towards the elderly patients.

There are some significances in this study. Understanding from the findings of this study can result in a better quality of health care and elderly nursing practice in the integrated nurse care service wards. This study can also serve as a valuable source of information that could improve the elderly nursing practice of nurses in the integrated nurse care service wards. Moreover, the findings of the study can contribute to the enrichment of the available literature on the elderly nursing practice of nurses in the integrated nurse care service wards, and on the health policies.

## Figures and Tables

**Table 1:** General characteristics of study participants.

Characteristics	*n* (%)	*n* (%)
Gender		
Female	228 (95.8)	204 (97.1)
Male	10 (4.2)	6 (2.9)
Age (year)		
<30	125 (52.6)	109 (51.9)
30~40	78 (32.7)	69 (32.8)
≥41	35 (14.7)	32 (15.3)
Mean ± SD	29.34 ± 6.82	29.87 ± 7.16
Education level		
College	108 (45.5)	96 (45.7)
Bachelor’s	115 (48.2)	102 (48.6)
Masters or above	15 (6.3)	12 (5.7)
Religion		
Atheists	131 (55.2)	115 (54.7)
Christians	73 (30.8)	65 (31.0)
Catholic	14 (5.8)	12 (5.7)
Buddhists	20 (8.2)	18 (8.6)
Marital status		
Single	184 (77.3)	161 (76.7)
Married	54 (22.7)	49 (23.3)
Total working experience (year)		
<5	138 (57.8)	121 (57.6)
≥5~<10	56 (23.4)	48 (22.8)
≥10~<15	28 (11.8)	26 (12.4)
≥15	16 (7.0)	15 (7.2)
Mean ± SD	5.87 ± 6.23	5.83 ± 5.57
Working experience of an INCSW (year)		
<0.5	44 (18.3)	39 (18.6)
≥0.5~<1	40 (16.8)	36 (17.2)
≥1~<2	49 (20.4)	40 (19.0)
≥2~<3	40 (16.8)	36 (17.1)
≥3~<4	39 (16.4)	36 (17.1)
≥4	26 (11.3)	23 (11.0)
Mean ± SD	1.97 ± 1.48	1.92 ± 1.53
Department ward		
Internal medicine	78 (32.8)	68 (32.4)
Surgical ward	103 (43.3)	90 (42.9)
Mixing ward	57 (23.9)	52 (24.7)
Job title		
General nurses	224 (94.2)	197 (93.8)
Charge or head nurses	14 (5.8)	13 (6.2)
Satisfaction with current workplace		
Satisfaction	87 (36.6)	76 (36.2)
Just so-so	129 (54.2)	113 (53.8)
Dissatisfaction	22 (9.2)	21 (10.0)
Level of salary (million won)		
<25	15 (6.2)	14 (6.7)
≥25~<35	152 (63.8)	135 (64.3)
≥35	71 (30.0)	61 (29.0)
Experience of living with the elderly		
Yes	90 (37.8)	80 (38.1)
No	148 (62.2)	130 (61.9)
Experience in psychiatric ward work		
Yes	9 (3.8)	7 (3.3)
No	229 (96.2)	203 (96.7)

INCSW: integrated nurse care service ward.

**Table 2:** Levels of empathy, self-efficacy, and elderly nursing practice.

Variables	Total Mean ± SD	Average Mean ± SD	Range of Tool (Average/Total)
Empathy	192.12 ± 14.86	3.26 ± 0.32	1–5/60–300
Self-efficacy	76.23 ± 7.42	3.21 ± 0.28	1–5/24–120
Elderly nursing practice	52.78 ± 7.62	3.28 ± 0.42	1–4/16–64

**Table 3:** Correlations among the study variables.

Variables	Empathy	Self-Efficacy	Elderly Nursing Practice	Age	Clinical Career	Working Experience of an INCSW
Empathy	1					
Self-efficacy	0.32	1				
Elderly nursing practice	0.27 *	−0.04	1			
Age	0.17 *	0.13 *	0.02	1		
Total working experience	0.16 *	0.08	0.03	0.67 *	1	
Working experience of INCSW	−0.02	0.03	−0.04	−0.03	0.07	1

INCSW: integrated nurse care service ward. * *p* < 0.05.

**Table 4:** Differences in elderly nursing practice according to the general characteristics of study participants (*n* = 210).

Characteristics	Elderly Nursing Practice		
	Mean ± SD	*t*/F	*p*
Gender		−0.52	0.723
Female	53.25 ± 6.55		
Male	53.76 ± 11.51		
Age (year)		0.48	0.649
<30	52.34 ± 7.22		
30–40	52.36 ± 7.85		
41–50	53.23 ± 7.54		
>50	49.48 ± 9.38		
Education level		0.10	0.887
College	52.88 ± 6.64		
Bachelor’s	52.23 ± 7.24		
Masters or above	53.25 ± 8.68		
Religion		1.21	0.423
Atheists	53.12 ± 7.46		
Christians	52.54 ± 6.69		
Catholic	49.67 ± 9.17		
Buddhists	51.88 ± 7.24		
Marital status		−0.38	0.745
Single	53.02 ± 7.21		
Married	53.25 ± 7.65		
Total working experience (year)		1.84	0.354
<5	52.84 ± 6.32		
≥5~<10	53.58 ± 6.15		
≥10~<15	51.63 ± 9.88		
≥15~<20	53.24 ± 6.74		
≥20	54.75 ± 7.42		
Working experience of an INCSW (year)		1.87	0.223
<0.5	55.23 ± 6.28		
≥0.5~<1	51.42 ± 6.44		
≥1~<2	52.32 ± 6.58		
≥2~<3	51.22 ± 7.23		
≥3~<4	50.54 ± 7.47		
≥4	53.87 ± 6.64		
Department ward		0.17	0.746
Internal medicine	53.02 ± 7.23		
Surgical ward	52.88 ± 6.46		
Mixing ward	52.76 ± 7.84		
Job title		0.58	0.432
General nurses	52.35 ± 6.72		
Charge nurses	51.87 ± 4.52		
Head nurse	55.23 ± 5.74		
Satisfaction with current workplace		1.38	0.312
Satisfaction	53.12 ± 6.28		
Just so-so	52.43 ± 6.56		
Dissatisfaction	52.24 ± 7.24		
Level of salary (million won)		0.68	0.762
<25	53.62 ± 8.76		
≥25~<35	53.58 ± 7.46		
≥35	53.43 ± 6.74		
Experience of living with the elderly		−1.13	0.275
Yes	52.43 ± 7.23		
No	51.88 ± 7.47		
Experience in psychiatric ward work		2.48	0.023 *
Yes	59.42 ± 6.74		
No	52.68 ± 7.22		

INCSW: integrated nurse care service ward, *t*/F: *t*-test/F test, SD: standard deviation, * *p* < 0.05.

**Table 5:** Influential factors relating to elderly nursing practice.

Variables	B	SE	β	*t*	*p*
Constant	31.73	6.84		4.24	<0.001
Empathy	0.16	0.05	0.30	4.21	<0.001 *
Self-efficacy	−0.15	0.07	−0.12	−1.73	0.064
Marital status	0.55	1.52	0.04	0.42	0.702
Total working experience	−0.02	0.02	−0.12	−0.62	0.524
Job title	1.38	1.54	0.08	0.68	0.418
Experience of living with the elderly	1.22	1.11	0.08	1.14	0.282

Adjusted *R*² = 0.32, F = 48.89, *p* < 0.001 *, Durbin-Watson = 1.78. B: unstandardized coefficients, SE: standard error, β: standardized coefficients, *t*: *t*-test, *p*: *p* value * *p* < 0.05.

## Data Availability

No new data were created or analyzed in this study. Data sharing is not applicable to this article.

## References

[1] Statistics Korea (2020). Future Population Estimation 2017–2067.

[2] National Health Insurance Korea (2019). Intergraded Nurse Care Service (2019).

[3] National Health Insurance Service Ilsan Hospital Korea (2019). Intergraded Nurse Care Service Analysis and Institutional Development Plan (2019).

[4] Buyuk E.T., Rizalar S., Güdek E., Güney Z. (2015). Evaluation of empathetic skills of nurses working in oncology units in Samsun, Turkey. Int. J. Caring Sci..

[5] Johnson C.E., Jilla A.M., Danhauer J.L. (2018). Didactic content and experiential aging simulation for developing patient-centered strategies and empathy for older adults. Semin. Hear..

[6] Lee G.E., Cho J.K., Ham S.H., Jeong M.Y. (2014). Nurses’ experiences in caring for elderly inpatients in a medical center. J. Korean Gerontol. Nurs..

[7] Hazif-Thomas C., Thomas P. (2015). Empathy fatigue and psychiatric care of the elderly. Soins. Gerontology.

[8] Song E.S. (2015). The Factors of Empathy for Older Patient in General Hospital Nurses.

[9] Van der Elst E., Dierckx de Casterlé B., Gastmans C. (2012). Elderly patients’ and residents’ perceptions of ‘the good nurse’: A literature review. J. Med. Ethics.

[10] Hong S.H., Park Y.H., Moon J.S. (2016). Relationship between moral sensitivity and elderly nursing practice of nurses. Korean J. Med. Ethics.

[11] Richter D., Kunzmann U. (2011). Age differences in three facets of empathy: Performance-based evidence. Psychol. Aging.

[12] Stone J.R. (2012). Elderly and older racial/ethnic minority healthcare inequalities care, solidarity, and action. Camb. Q. Healthc. Ethics.

[13] Seo N.S., Moon J.S., Hong S.H., Park Y.H. (2016). The influence of attitude, experience and empathy on the nursing care of the elderly who have no caregiver. Korean J. Health Serv. Manag..

[14] Lee E., Chang S.S. (2016). Factors influencing depression of nurses among comprehensive nursing care service ward. Korean J. Occup. Health Nurs..

[15] Lee M.K., Jung D.U. (2015). A study of nursing tasks, nurses’ job stress and job satisfaction in hospital with no guardians. J. Korean Acad. Nurs. Adm..

[16] Bandura A. (1986). Social Foundation of Thought and Action: A Social Cognitive Theory.

[17] Ko Y.Y., Kang K.H. (2006). A study on the relationship between self-efficacy collective-efficacy and job stress in the nursing staff. J. Korean Acad. Nurs. Adm..

[18] Faul F., Erdfelder E., Lang A.G., Bunchner A. (2007). G power 3: A flexible statistical power analysis program for social, behavioral, and biochemical sciences. Behav. Res. Method.

[19] Monica E.L. (1981). Construct validity of an empathy instrument. Res. Nurs. Health.

[20] Park E.S., Suk M.H., Jung K.S. (1997). A study on the empathy of pediatric nurses. Child Health Nurs. Res..

[21] Kim A.Y., Cha J.E. (1996). Self-efficacy and measurement. Korean J. Ind. Organ. Psychol..

[22] Choi H.J. (2002). The Research is Nurse of Knowledge, Attitude and Practice for Old Age Patient.

[23] Yeun Y.R. (2015). Effects of comprehensive nursing service on the nursing performance, job satisfaction and customer orientation among nurses. J. Korea Acad. Ind. Coop. Soc..

[24] Lee K.J., Park H.J., Kim S.S. (2014). A comparative study on the counseling self-efficacy and empathy of psychiatric nurses and general ward nurses. Health Nurs..

[25] Kim S., Jeong S.H., Lee M.H., Kim H.K. (2017). Importance, performance and rates of nurse performance of nursing interventions in long-term care hospitals. J. Korean Acad. Nurs. Adm.

[26] Park S.E. (2011). Nursing Activities and Delegation Status of Registered Nurses in Geriatric Hospital.

[27] Kang H.S., Sung K.W. (2015). Influence of emotional intelligence and organization commitment on geriatric nursing practice of nurses in long-term care hospital. J. Korean Gerontol. Nurs..

[28] Kwon Y.H., Lee H.Y., Hwang S.S. (2013). A study on the knowledge, attitude and nursing practice of the nurse-towards the elderly in geriatric hospitals. J. Korea Acad. Ind. Coop. Soc..

